# Wood density can best predict carbon stock in the forest aboveground biomass following restoration in a post open limestone mining in a tropical region

**DOI:** 10.3389/fpls.2025.1553886

**Published:** 2025-02-20

**Authors:** Junyang Mao, Peipei Xue, Yuxin Chen, Ting Xiang, Hui Zhang, Cui Chen, Qingqing Yang, Wenfeng Gong

**Affiliations:** ^1^ School of Ecology, Hainan University, Haikou, China; ^2^ Chongqing Academy of Forestry, Wulingshan Forest Eco-station, Chongqing, China; ^3^ Wanning Tropical Lowland Rainforest Restoration and Utilization, Hainan Observation and Research Station, Haikou, China; ^4^ School of Geography and Tourism, Huanggang Normal University, Huanggang, Hubei, China; ^5^ Hainan Academy of Forestry (Hainan Academy of Mangrove), Haikou, China

**Keywords:** carbon emission, carbon stock, functional traits, land use, vegetation

## Abstract

**Introduction:**

Reforestation has been widely considered to best solve this problem, but this requires an accurate estimation of carbon stocks in the forest aboveground biomass (AGB) at a large scale. AGB models based on traits and remote sensing indices (moisture vegetation index (MVI)) are the two good methods for this purpose. But limited studies have developed them to estimate carbon stock in AGB during restoration of degraded mining areas.

**Methods:**

Here, we have successfully addressed this challenge as we have developed trait-based and MVI-based AGB models to estimate carbon stock in the AGB after performing reforestation in a 0.2 km^2^ degraded tropical mining area in Hainan Island in China. During this reforestation, seven non-native fast-growing tree species were planted, which has successfully recovered soil processes (including soil microorganisms, nematodes and chemical and physical properties).

**Results and discussions:**

By using these two models to evaluate carbon stock in AGB, we have found that an average of 78.18 Mg C hm^-2^ could be accumulated by our reforestation exercise. Moreover, wood density could predict AGB for this restored tropical mining site, and indicated that strategies of planting fast-growing species leads to fast-growing strategies (indicated by wood density) which in turn determined the largely accumulated carbon stocks in the AGB during restoration. This restoration technology (multiple-planting of several non-native fast-growing tree species) and the two accurate and effective AGB models (trait-based and MVI-based AGB models) developed by us could be applied to 1) restore other degraded tropical mining area in China, and 2) estimate carbon stock in forest AGB after performing restoration.

## 1 Introduction

Variations in global carbon stocks are largely determined by terrestrial ecosystem (Migliavacca et al., 2021; Tang et al., 2022). Globally, industrial mining has destroyed nearly 2 million hectares of land (FAO, 2015), and the changes in land use and cover as a consequence of mining are considered as a main driver of terrestrial carbon loss (Baier et al., 2022; Tagesson et al., 2020). Mining activities have influenced approximately 11.5% of the global terrestrial area (Luckeneder et al., 2021). Thus, ecological degradation and emission of greenhouse gases throughout the world may be aggravated by mining, which in turn influence the global climate and pose a serious threat to the ecological safety (Zhu et al., 2024).

Two potential ways have been assumed to be effective in balancing the global C cycle. The first involves cutting the carbon emissions, whereas the second warrants an increase in the natural C sink to offset the increased carbon emissions (Ahirwal et al., 2017). The loss of forest-cover due to open strip mining activities have significantly increased the C level in the atmosphere (Mukhopadhyay and Maiti, 2014). Increasing forest area could be a sustainable tool to mitigate elevated atmospheric CO_2_ concentration (IPCC, 2001). As a result, performing reforestation in degraded mining area to increase forest cover has widely been suggested to decrease the potential carbon emission due to mining (Ahirwal et al., 2017; Yuan et al., 2023; Zhu et al., 2024). Tropical forests contain 55% of the global stores of aboveground forest carbon (Pan et al., 2011; Philipson et al., 2020). Mining constitute one of the biggest threats to vegetation and soil in the tropical forests, which thereby gives rise to a large amount of carbon emission (Ahirwal et al., 2017; Zhang et al., 2024a). Thus, it is very necessary to perform reforestation in degraded tropical mining area to enlarge natural C sink to prevent the increase in the atmospheric CO_2_ level.

Accurate estimation of forest aboveground biomass (AGB) can directly determine the C accumulation capacity of the tree species in the restored mining area. The greater estimates of AGB indicate a high C accumulation capacity of the plants (Zhao et al., 2014; Ahirwal et al., 2017). Traditionally, AGB is estimated by harvesting multiple individuals of several tree species to obtain diameter at breast height (DBH) and height (H) for developing an estimation model [AGB = a × (DBH^2^ × H^b^)] (Chave et al., 2005). Then, by measuring DBH and H for all the individuals of every tree species in an ecosystem could accurately estimate the AGB for an ecosystem. However, this method could only be used at a small scale, because it takes a long time to measure DBH and H of all trees of every species in a forest.

Two more ABG models could be developed for a forest ecosystem, (1) a remote sensing indices-based AGB model; and (2) a trait-based AGB model. Vegetation indices obtained from remote sensing (moisture vegetation index (MVI)) are highly related to forest AGB in tropical forests (Boyd, 1999; Foody et al., 2003; Freitas et al., 2005). MVI, at large and regional scales, could be easily obtained from remote sensing images. Therefore, MVI-based model is more effective than a traditional model for estimating AGB for a forest ecosystem. Functional traits that are correlated with the growth rate of individual plants (Perez-Harguindeguy et al., 2013) are also expected to be mechanistically related to primary productivity of the vegetation (Garnier et al., 2004). It has been found that key traits (for example, specific leaf area (SLA), and wood density) not only directly determine AGB, they also indicate the mechanisms that result in alterations in AGB in the tropical forests (Baker et al., 2004; Nam et al., 2018; Phillips et al., 2019; Finegan et al., 2015; Wang and Ali, 2021). Functional traits could only be measured for 3-5 individuals of one tree species (Garnier et al., 2004; Zhang et al., 2018). Consequently, compared to a traditional model, a trait-based model is also more effective for the estimation of AGB for tropical forests. However, relatively few studies have developed these two models to accurately and effectively estimating AGB for tropical forests, let alone for restored tropical mining area.

**Figure 1:**
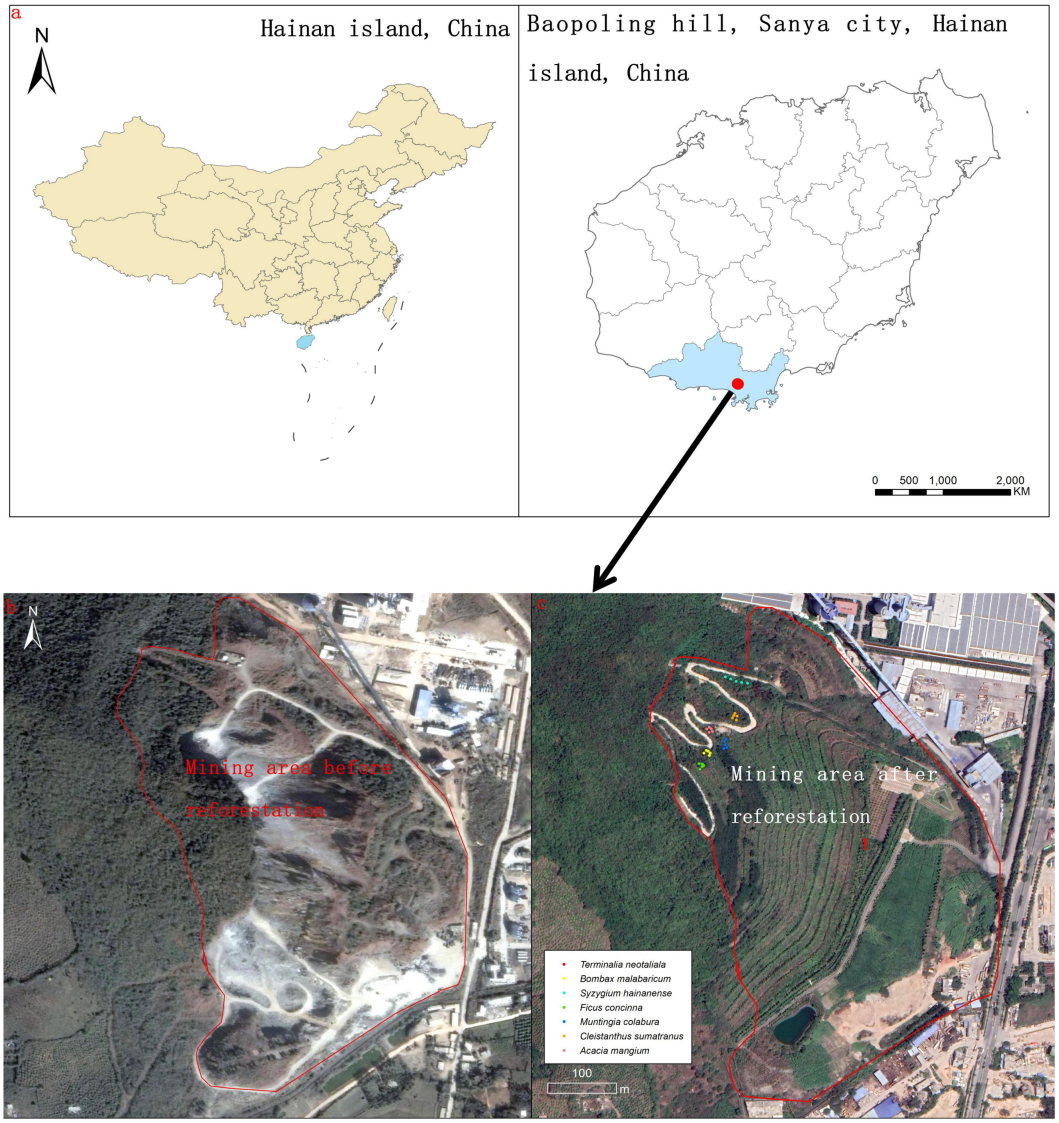
The location of the study sites **(A)**, landscape for mining area before and after reforestation, and the location of our sampled 35 trees of the seven species (5 individuals for each tree species) that were used for our reforestation and generating the aboveground biomass (AGB) model **(B, C)**.

Since 2013, a reforestation project has been initiated to restore a 0.2 km^2^ degraded tropical limestone mining area near the southern edge of Hainan Island (**Figures 1A–C**, Zhang et al., 2024a, b). Limestone mining for the cement industry had been performed for two decades in this area, which had converted an original tropical rainforest into an open-mining area (bare rocky substrates, without any plants; **Figures 1B, C**, Zhang et al., 2023, 2024a). By mix-planting one non-native shrub species and seven non-native fast-growing tree species, this open-mining area now has been successfully restored into a secondary tropical rainforest, whose soil microorganisms, nematodes and physical and chemical properties are comparable to those of an adjacent undisturbed tropical rainforest (Zhang et al., 2024a, b).

Limestone mining and the global production of cement has grown in tandem with economic development (Uwasu et al., 2014; Farfan et al., 2019). The exponential increase in cement production and export in recent decades has resulted in a global annual quarrying of over 178 million metric tons, with huge areas being mined for limestone, particularly in Asia (Hughes et al., 2017). The production of cement at this scale has enormous environmental implications as it utilizes about 1.9% of the global electricity production and contributes 5-8% of the global CO_2_ emissions (Farfan et al., 2019).

Moreover, China has become a major mining country, which in turn may lead it to be one of the largest carbon emitters (assessment of carbon sequestration potential of mining areas under ecological restoration in China). Recovering the degraded mining areas, preventing the geological hazards and performing reforestation to recover the green landscape in the mining areas are the main focus in China (Chen et al., 2022; Zhu et al., 2024). However, it remains unclear whether restored mining areas could increase carbon sequestration potential (assessment of carbon sequestration potential of mining areas under ecological restoration in China). Thus, the above-mentioned restored tropical limestone mining area (in the Hainan Island) provide us with a perfect platform to develop trait-based and MVI-based AGB models to determine the amount of carbon accumulated by this successful reforestation. For achieving this goal, we first harvested a total of 35 trees (five individuals for each of the seven native tree species) to obtain DBH and H so that we could develop a traditional AGB model. Then, we measured six key functional traits (transpiration rate (mmol m^−2^ s^−1^), stomatal conductance (mmol m^−2^ s^−1^), leaf hydraulic conductance (mmol m^−1^ s^−1^ MPa^−1^), photosynthesis rate (μmol m^−1^ s^−1^), specific leaf area (cm g^-1^) and wood density (g m^-3^) and obtained the MVI from the remote sensing images for this restored tropical mining area. Finally, we utilized data on six functional traits, and the MVI to further generate more effective trait-based and MVI-based AGB models. These two models could not only accurately estimate how much carbon stock was accumulated by our reforestation, but also provide two useful tools for estimating AGB for other restored tropical mining areas in China.

## 2 Materials and methods

### 2.1 Study sites

The study site was located in a limestone mountain near Sanya City of Hainan Island, China (110°58′01′′E, 19° 38′48′′N; Baopoling Mountain; 300 m a.s.l). The area has a tropical monsoon oceanic climate, with a mean annual temperature of 28°C, and 1500 mm of mean annual precipitation, about 91% of which occurs between the months of June to October (Zhang et al., 2024a). The natural vegetation of the area is classified as broadleaf tropical rainforest (Zhang et al., 2024a). In this area, limestone was extensively mined between 1995 to 2015 (Zhang et al., 2023). We reforested a degraded mine area of about 0.2 km^2^, whose mining history and our reforestation efforts has been described in detail in a recent study (Zhang et al., 2023).

### 2.2 Harvesting of trees and sampling of functional traits in the restored mining area

Since seven non-native tree species (*Terminalia neotaliala*, *Bombax ceiba*, *Ficus concinna*, *Muntingia colabura*, *Cleistanthus sumatranus*, *Acacia mangium* and *Syzygium hainanense*) were used in our restoration, we harvest 35 trees (five individuals for each species). First, we measured the DBH and height for all the individuals for all the seven tree species in the restored site and calculated the mean DBH and height for each of the seven non-native tree species. Then, for each species, we randomly selected 5 individuals whose DBH and height were comparable to the mean DBH and height; their locations were shown in detail in **Figure 1C**. Finally, we harvested the aboveground parts of all 35 selected trees and obtained their dry weight as AGB. We also used these 35 selected trees/individuals to measure six functional traits (transpiration rate, stomatal conductance, leaf hydraulic conductance, photosynthesis rate, specific leaf area and wood density). The details of measurements of these six functional traits have been described in previous studies (Li et al., 2015; Shen et al., 2016; Zhang et al., 2018), and further summarized in the **Supplementary Material**.

### 2.3 The developments of traditional, trait-based and MVI-based AGB models

Following Chave et al. (2005), we first used the following equation (AGB= a×(DBH^2^×H)^b^) to develop a traditional AGB model. Then, we used a penalization on the number of parameters, the Akaike information criterion (AIC) to get the best parameter (a and b). Specifically, the best a and b should be derived at the minimum AIC. We also provided the residual standard error (RSE) to be used as an alternative statistic.

For developing a MVI-based AGB model, we first collected the remote sensing images for the restored mining areas, which are from Landsat8 OLI (http://glovis.usgs.gov/) and Jilin-1 (https://www.jl1mall.com/store/). The ENVI software was used to perform a series of processing, such as radiometric correction, atmospheric correction, and terrain correction, for the remote sensing images of the mining areas from Landsat8 OLI. The ENVI software was also first used for completing the radiometric and atmospheric corrections for Jilin-1. Then, topographic maps (1:10000), the RTK sampling of the ground point coordinates, and the DEM data for the whole of the restored mining area were utilized to finish the orthorectified processing of Jilin-1, with a resampling resolution of 0.75m. By using the Jilin-1 image as the reference, automatic registration module in the ENVI software was used to complete the dynamic matching processing of Landsat8 OLI and Jilin-1 remote sensing images, and the fusion processing of Landsat8 OLI and Jilin-1 was completed to generate the high-resolution remote sensing data, whose map projection coordinate system was WGS1984-UTM_Zone49N. Following Freitas et al. (2005), this study selected the following combinations of red band (0.63-0.69 μ m) and short infrared band (1.55-1.75 μ m) to analyze the generated high-resolution remote sensing data to get the moisture vegetation index (MVI). The following equation was used: MVI5=(NIR−MIR5)/(NIR+MIR5) , where NIR and MIR5 represent the near-infrared band and mid-infrared band, respectively. Specifically, the unit for unit size for extracting the Moisture Vegetation Index (MVI) is based on each 0.75 ×0.75 m^2^ image. With the support of ArcGIS Pro software, the mean DBH and H for the mining area were used to develop the model between MVI and DBH, and between MVI and H. Specifically, the DBH-MVI and H-MVI models are DBH=1.1770×MVI5_m_00 + 11.7964 and H=44.171×MVI5_r_01-10.06405 and respectively (**Supplementary Figures S1 and S2**). Finally, the MIV-based AGB model was directly obtained by using the developed traditional AGB mode.

In terms of trait-based AGB model, we only used the linear regression to quantify the relationship between AGB and each of the six functional traits (transpiration rate, stomatal conductance, leaf hydraulic conductance, photosynthesis rate, specific leaf area and wood density). Our main purpose was to find out which trait could best determine AGB.

## 3 Results

**Figure 2:**
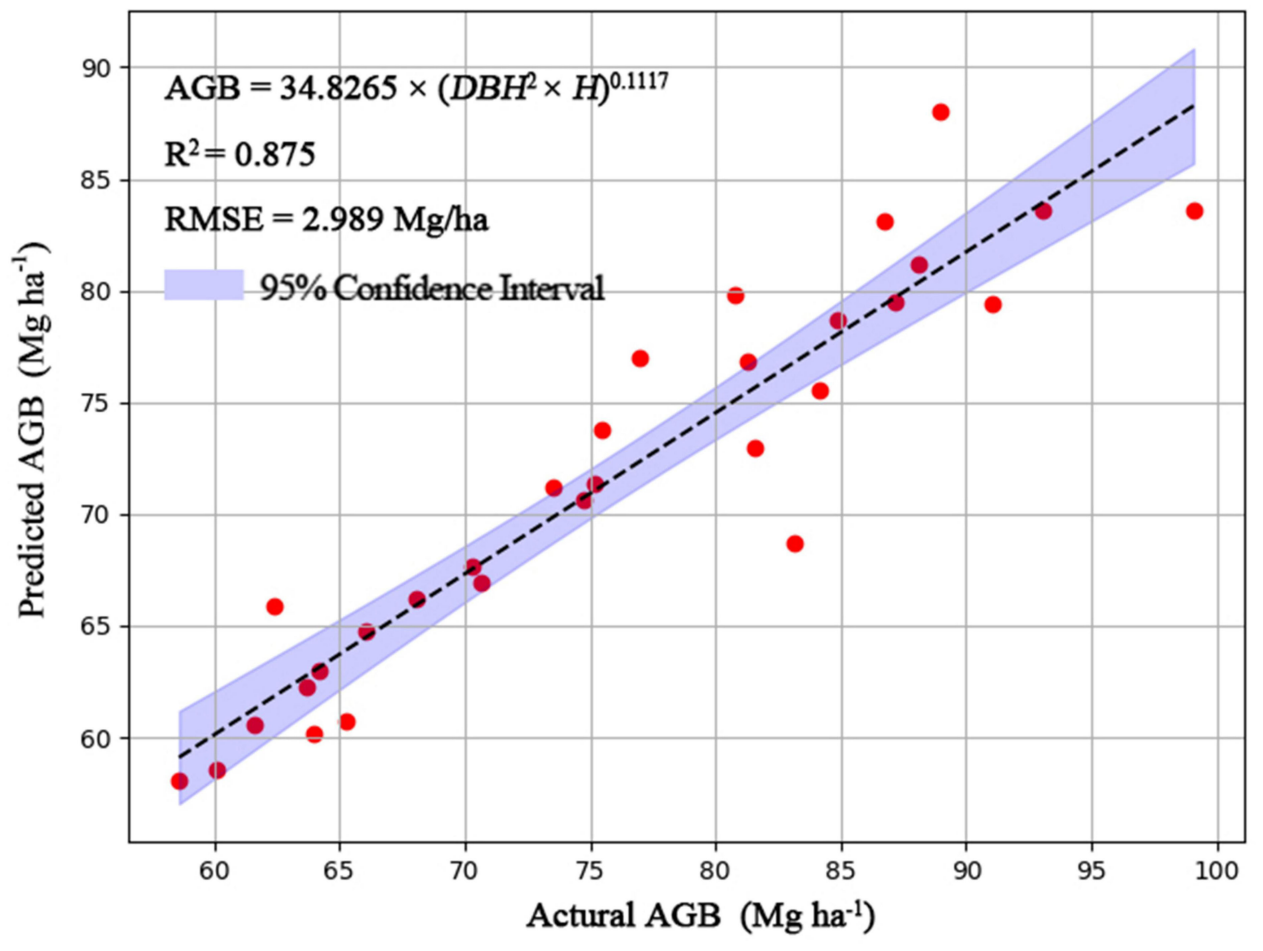
Development of a traditional AGB model (AGB= 34.8279×(DBH^2^×H)^0.1117^) on the basis of AGB, DBH and height for our sampled 35 individuals for the seven tree species.

**Figure 3:**
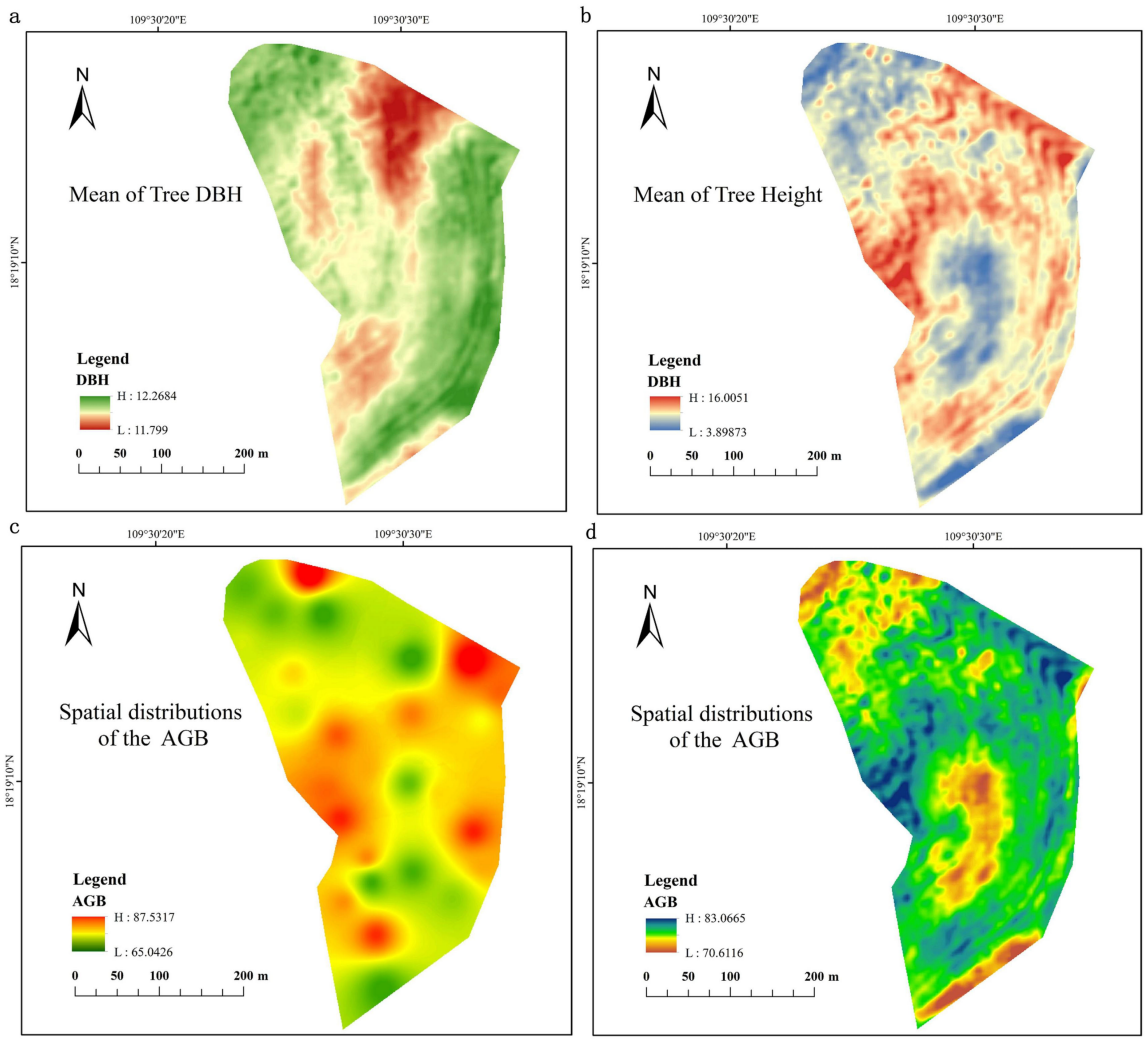
Predicted mean height **(A)** and DBH **(B)** that were used along with the remote sensing images. Actual AGB **(C)** and predicted AGB **(D)**, which are based on traditional AGB model, are presented.

**Figure 4:**
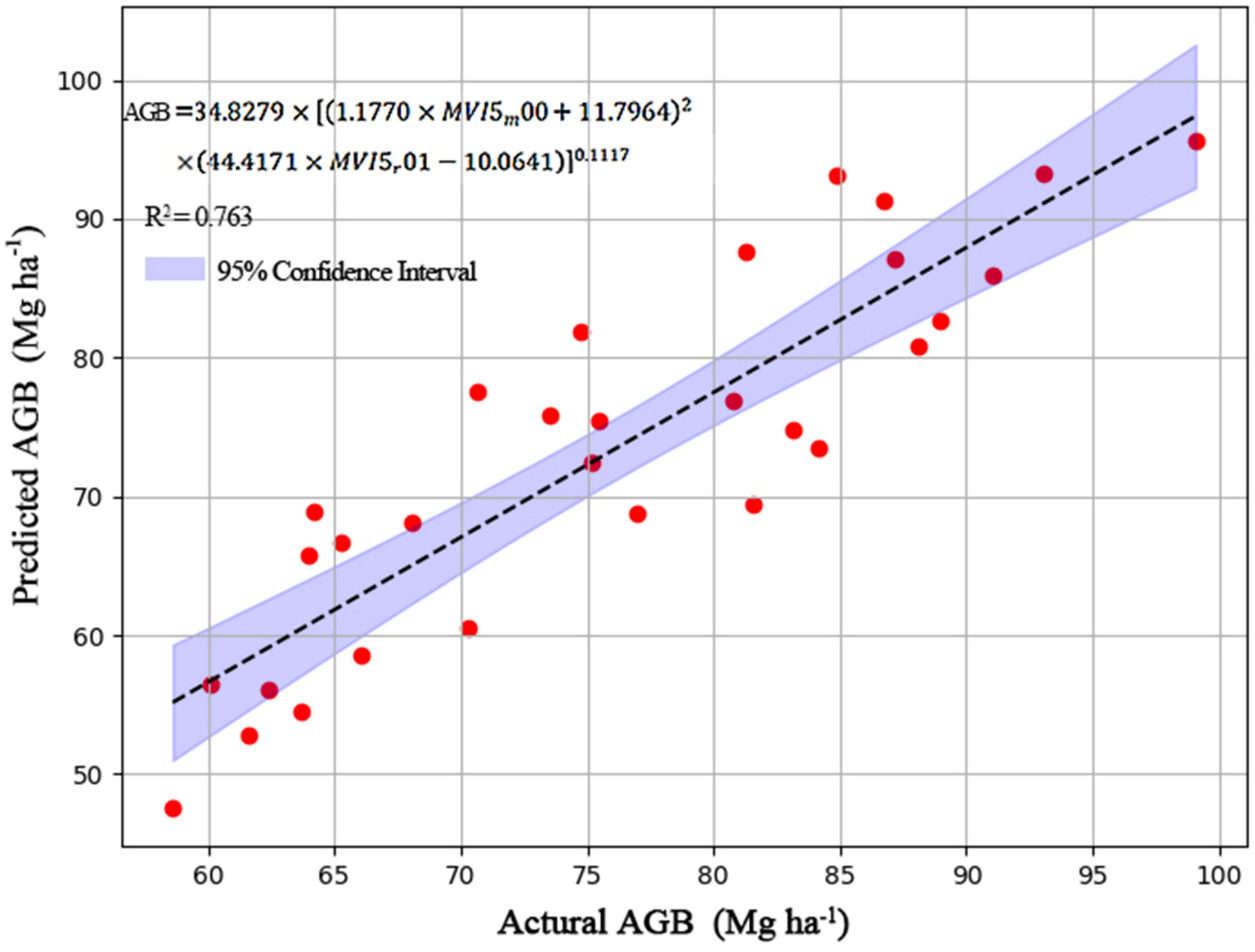
Development of a MVI-based AGB model (AGB= 34.8279×((1.770×MVI5_m_00 + 11.7964)^2^×(44.4171×MVI5_r_01-10.0650511.7964))^0.1117^).

By using the measured mean DBH and H values for the restored mining area, and AGB for the harvested 35 individuals for the seven tree species, we finally derived the traditional AGB model as following: AGB= 34.8279×(DBH^2^×H)^0.1117^ (**Figure 2**). By using the measured mean DBH and H for the restored mining area and the generated high-resolution remote sensing data, which is derived from Landsat8 OLI and Jilin-1, first, we got the model between the DBH and MVI (DBH=1.770×MVI5_m_00 + 11.7964; the generated mean DBH of the whole mining area is presented in **Figure 3A**). Also, we obtained a model between H and MVI as (H=44.4171×MVI5_r_01-10.0650511.7964; the generated mean H for the whole of the mining area is presented in **Figure 3B**). Here, MVI5_m_00 and MVI5_r_01 represent the mean MVI in the wet and dry season, respectively. Then, by using the developed traditional AGB model, we finally obtained the MVI-based AGB model as (AGB= 34.8279×((1.770×MVI5_m_00 + 11.7964)^2^×(44.4171×MVI5_r_01-10.0650511.7964))^0.1117^, **Figure 4**). We also provided the actual AGB (ranging from 65.0426-87.5317, **Figure 3C**) and predicted the AGB (ranging from 70.6116-83.0665, **Figure 3D**), which are based on both traditional and the MVI-based AGB models.

**Figure 5:**
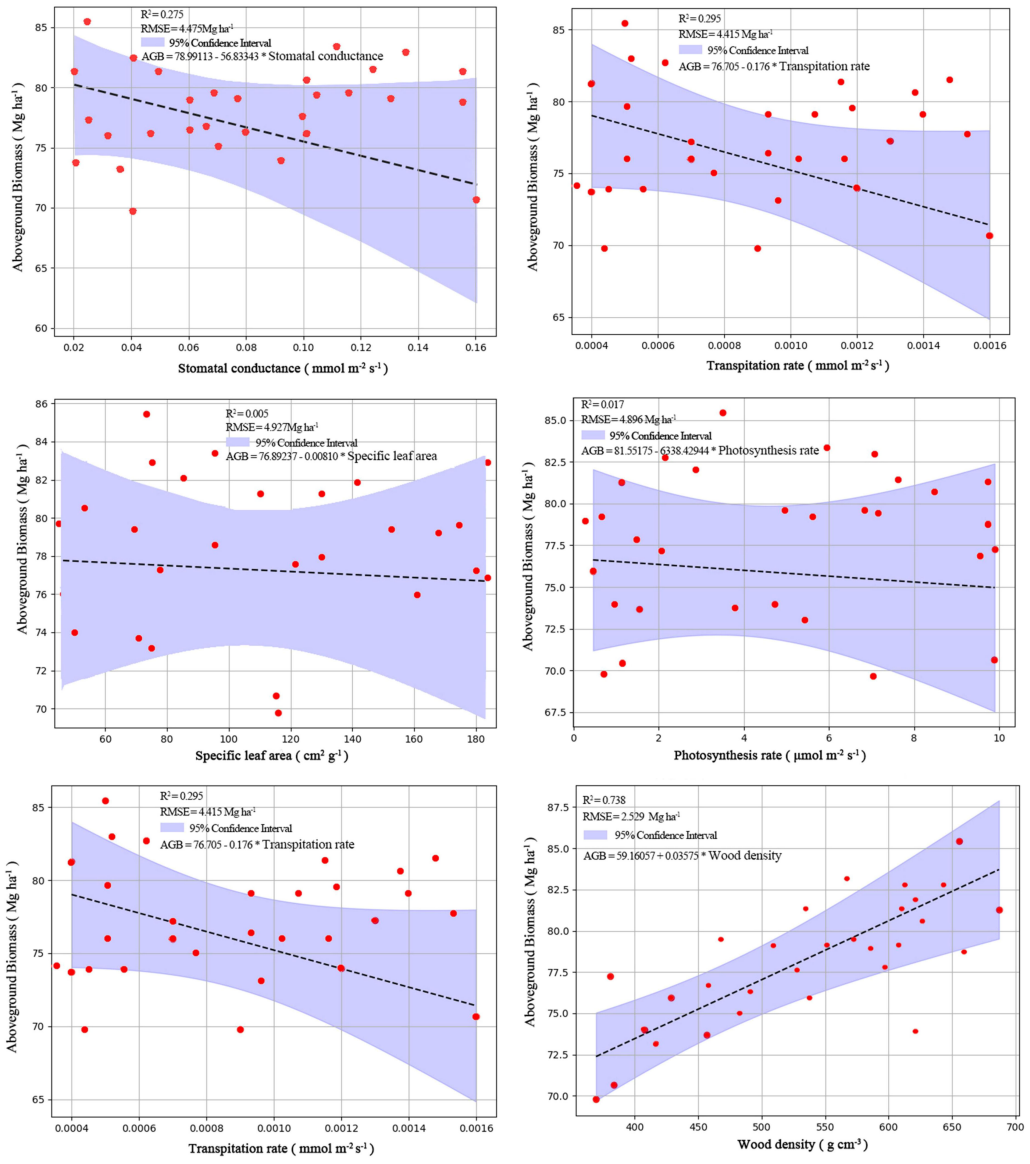
The relationships between the six functional traits (transpiration rate, stomatal conductance, leaf hydraulic conductance, photosynthesis rate, specific leaf area and wood density) and AGB. Specifically the responding trait-based model for these six traits are shown respectively as: AGB=59.16057 + 0.03575×WD (wood density), AGB=76.705-0.176×A (transpiration rate), AGB=76.89237 + 0.00810×SLA (specific leaf area), AGB=85.55175 + 6338.4294×E (photosynthesis rate), AGB=78.99113 + 56.83343×gsw (stomatal conductance), and AGB=81.98104 + 435.44942×Kleaf (leaf hydraulic conductance).

Our linear regression analysis clearly demonstrated that among the six functional traits, only wood density could determine the AGB. Wood density was significantly positively related to AGB (**Figure 5**), whereas the other five traits were not significantly associated (**Figure 5**).

## 4 Discussion

A greater AGB indicates a high C accumulation capacity of the plants (Zhao et al., 2014; Ahirwal et al., 2017; Zhu et al., 2024). Developing trait-based and MVI-based AGB models could directly indicate the amount of C that could be accumulated during the reforestation of degraded tropical mining areas. In this study, we have successfully developed trait-based and MVI-based AGB models for accurately estimating the carbon stock in the AGB of a successfully restored 0.2 km^2^ tropical limestone mining area. By using these two models, we have found that an average of 78.18 Mg C hm^-2^ could have been accumulated during our reforestation exercise. This value is much higher than those in the other restored tropical mining areas. For example, Ahirwal et al. (2017) showed that a restored tropical mining area could only accumulate 23.7 Mg C hm^-2^. Similarly, Agus et al. (2016) had demonstrated that recovered tropical mining area was able to accumulate less than 30 Mg C hm^-2^. It has been found that mature tropical forests can accumulate 57-375 Mg C hm^−2^ across the tropics (Lewis et al., 2013; Niiyama et al., 2010). In these scenarios, our reforestation exercise was able to achieve high levels of accumulated carbon stocks. Consequently, we recommend that our tropical limestone mining restoration program should be expanded to other degraded tropical limestone mining areas.

Vegetation indices (MVI) has been widely used to estimate AGB at large scale (Freitas et al., 2005). But to the best of our knowledge, limited studies have developed a MVI-based model for estimating AGB in a restored tropical mining area. By combining the use of a traditional AGB model and a MVI obtained from remote sensing images, for the first time, our study has successfully developed a MVI-based AGB model (AGB= 34.8279×((1.770×MVI5_m_00 + 11.7964)^2^×(44.4171×MVI5_r_01-10.0650511.7964))^0.1117^). This model can be applied to estimate the amount of carbon that has been stored after the reforestation of the degraded tropical mining areas.

The relationships between the six traits and the AGB clearly demonstrated that only wood density could predict AGB for our restored limestone mining area. This result is consistent with other findings (Iida et al., 2012; King et al., 2006; Poorter et al., 2008). This also further confirms that wood density can determine AGB for tropical forests (Baker et al., 2004; Nam et al., 2018; Phillips et al., 2019). We found wood density is significantly positively related to AGB in our restored tropical mining area. This observation supports the findings in Chave et al. (2008), who show that the species with higher wood densities could attain more AGB than those with low wood density in different forest sites in three continents (Africa, America and Asia). Wood density is considered as a key trait for capturing growth of tropical forest species, with higher wood density indicating fast-growing strategies (Nam et al., 2018). Thus, this results also clearly elucidated fast-growing strategies determined the accumulation of C in all the tree species. The other five functional traits are highly associated with plant photosynthesis and hydrology (Li et al., 2015; Shen et al., 2016; Zhang et al., 2018). However, they were not good predictors of AGB in our restored tropical mining area. Thus, strategies of plant photosynthesis and hydrology may not be the good predictor of C accumulation in our restored tropical mining area. However, future control experiments of light and water environments are required to further verify this.

The choice of tree species for initial planting are primarily based on the goals of the restoration (de Souza Barbosa et al., 2021). But a natural recommendation is to plant native species to recover original biodiversity and function (Cunningham et al., 2015). Importantly, it has been amply demonstrated that restoration could contribute greatly to achieving the multiple sustainable development goals (SDGs) of the United Nations (Ahirwal and Maiti, 2022; Ghosh and Maiti, 2021a, b). Land supports the resources and provides the matrix for achieving multiple SDGs, which include climate action, life on land, reduction of poverty and hunger, human health and wellbeing, as well as affordable and clean energy (Ghosh and Maiti, 2021a). So, the goals of restoration could be very broad and comprehensive. Irrespective of the set of goals and the choice of tree species for initial planting, the basic ecological processes of colonization and establishment by native species, and the natural ecological successions are bound to operate and contribute to plant community development (Ahirwal et al., 2017).

Generally when the reforestation begins at an open-site conditions, fast-growing, and native early successional species are a natural choice for their better growth and survival and also their facilitation for recovering original biodiversity and function (Nunes et al., 2020; Reid et al., 2015). However, the choice of species may often be limited by the availability of seeds and seedlings for planting (Nunes et al., 2020; Osorio-Salomón et al., 2021). Therefore, non-native tree species have often been used in the initial planting for restoration (Cunningham et al., 2015; Damptey et al., 2020; Nunes et al., 2020). Planting non-native species may result in a poor or slow recovery of attributes like biodiversity, soil physical and biological structure (for example, soil bulk density and microorganism), soil water content, and soil fertility (Cunningham et al., 2015; Liu et al., 2021; Zhao et al., 2021). However, non-native species could greatly improve the recovery of biomass at a speed at which restoration can be achieved. Based on this scenario, we finally mix-plant seven non-native fast-growing tree species, to restore our degraded tropical mining area. This type of restoration has successfully restored this degraded tropical mining area into a secondary tropical rainforest, whose soil microorganisms, nematodes and physical and chemical properties are comparable to those of an adjacent undisturbed tropical rainforest (Zhang et al., 2024a, b). More important, we further demonstrate that this type of reforestation may result in very high carbon accumulation. As a result, multiple non-native fast-growing tree species should be utilized to perform restoration of degraded tropical mining area in China. However future comparison experiments should be performed whether planting native tree species can achieve more carbon stock than planting-non-native tree species.

## 5 Conclusion

The restoration of mined areas has been widely assumed as a good way to reduce carbon emission that result from mining. By using a successfully recovered tropical limestone mining platform, we have demonstrated that this restoration could indeed accumulate high levels of C, and thereby could facilitate in reducing C emissions. This restoration technology, along with the two accurate (and effective) AGB models (trait-based and MVI-based AGB models) developed in this study, can be applied to 1) restore other degraded tropical mining area in China, and 2) to evaluate carbon stocks in the forest AGB in other restored mining area.

## Data Availability

The raw data supporting the conclusions of this article will be made available by the authors, without undue reservation.
