# Supplementary material for: Wood density can best predict carbon stock in the forest aboveground biomass following restoration in a post open limestone mining in a tropical region
Source: Front Plant Sci. 2025 Feb 20;16:1553886. doi: 10.3389/fpls.2025.1553886 (PMC11882525; doi:10.3389/fpls.2025.1553886)

**Text S1.** Methods for functional trait measurements

## Measurements of photosynthesis rate, transpiration rate and stomatal conductance were conducted between 9:00 and 11:00 on sunny days with a Li-6400 portable photosynthesis system (Li-6400, Li-Cor, Lincoln, Nebraska, USA). Based on preliminary trials, photosynthetic photon flux density was set at 1500 μmol m^-2^ s^-1^ to ensure that light-saturated photosynthetic rates were measured for all species. Ambient CO_2_ and air temperature were maintained at 390 μmol mol^-1^ and 28°C, respectively. Before data were recorded, leaves were exposed to the above conditions for about 5 minutes to allow photosynthetic parameters to stabilize. Three to five mature individuals were selected and five to six fully expanded and sun-exposed leaves were sampled from each individual for the measurements of photosynthesis rate, transpiration rate and stomatal conductance.

## A total of ten healthy and leaf-bearing branches (6–8 mm in diameter) from three to five mature individuals for each species were cut off in early morning, sealed in black plastic bags with moist towels, and transported to the laboratory immediately. Before measurement, all of the branch samples were re-cut under water, and the cut ends were trimmed with a razor blade. The branch segments used in experiments were about 40-50 cm long. To remove air embolisms, branch segments were perfused with a filtered (diameter; 0.2 μm) 20 mmol KCl solution at a pressure of 0.1 MPa for 20 min. Each segment was then connected to a hydraulic conductivity-measurement apparatus following the method in . An elevated water reservoir supplied the same perfusion solution to the segment, with a head pressure of about 6 KPa. Water flow through the segment was allowed to equilibrate for about 10 min, after which the mass of water flux though the segment over time (in seconds) was measured. Maximum hydraulic conductivity of the segment (k_h_) was calculated as:

$k_{h}=\frac{F\times L}{P}$ (1)

where F was the flow rate (kg s^-1^), L was the length of the segment (m) and P was the pressure gradient (MPa) through the segment. K_s_ was equivalent to k_h_ divided by the mean value of sapwood cross-sectional area of both ends of the branch segment.

Leaves were collected from the six smallest and six largest individuals of each species. Leaf size (cm^2^) was determined using a scanner (CanoScan LiDE 700F), and analyzed with an image processing software (ImageJ, version 1.43u, National Institute of Mental Health, Bethesda, Maryland, USA). Leaves were dried at 60˚C for 72 h, and weighed to determine leaf dry weight. Specific leaf area was calculated as leaf size per unit of dry leaf mass.

Wood samples were collected from six randomly chosen individuals outside the plot. An increment borer was used to extract 1 cm diameter cores at approximately 1.5 m height on the main stem for trees larger than 6 cm in DBH, but for trees and shrubs < 6 cm height, we collected 10 cm long stem segments of 1 cm diameter from terminal branches. Wood density was calculated as the ratio of dry weight and fresh volume. We used water displacement method to determine fresh volume and drying at 60˚C for 96 h to determine dry mass.

# References

Sperry, J.S., Donnelly, J.R. & Tyree, M.T. (1988) A method for measuring hydraulica conductivity and embolism in xylem. *Plant Cell & Environment,* **11,** 35-40.

**Fig. S1.** Development of a diameter at breast height (DBH_r_)-MVI model (DBH_r_=1.1770×MVI5_m_00+11.7964) that were used along with the remote sensing images.


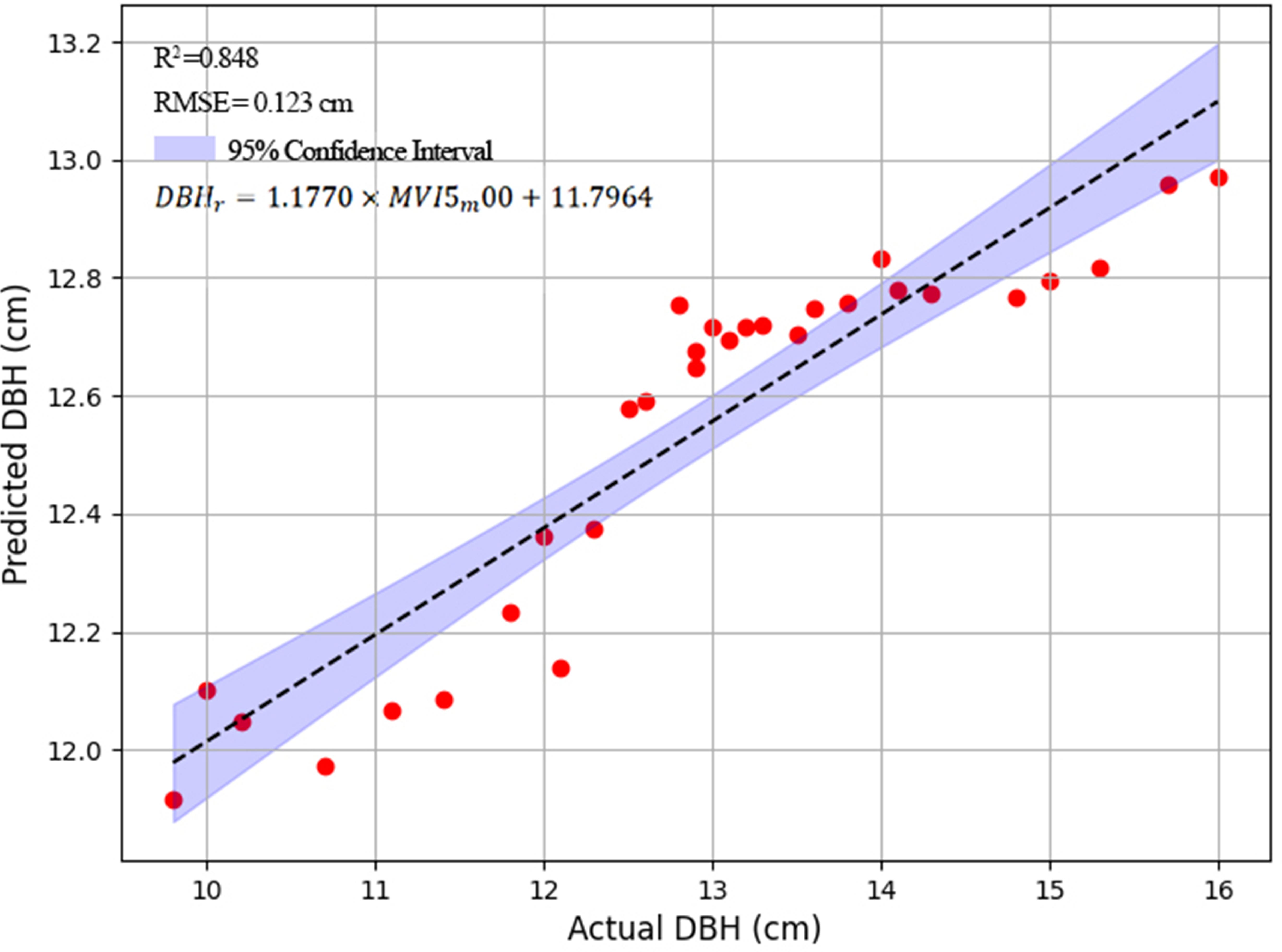


**Fig. S2.** Development of a tree height (H_m_)-MVI model (H_m_=44.171×MVI5_r_01-10.06405) that were used along with the remote sensing images.


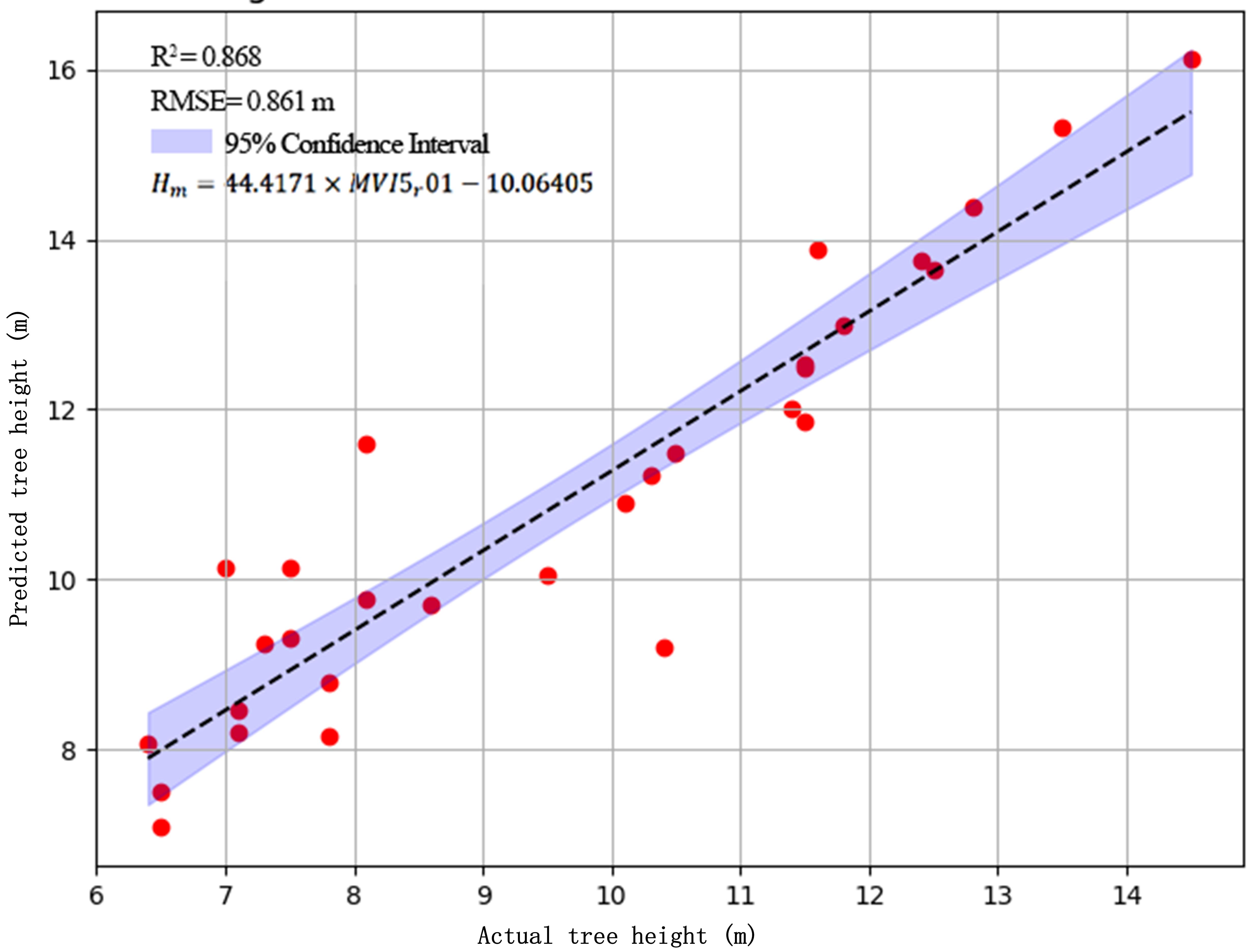

Supplement: Supplementary file 1 [file DataSheet1.doc]
